# Comparative transcriptome analysis of leaves during early stages of chilling stress in two different chilling-tolerant brown-fiber cotton cultivars

**DOI:** 10.1371/journal.pone.0246801

**Published:** 2021-02-09

**Authors:** Shouwu Tang, Yajie Xian, Fei Wang, Cheng Luo, Wu Song, Shuangquan Xie, Xifeng Chen, Aiping Cao, Hongbin Li, Haifeng Liu

**Affiliations:** 1 Key Laboratory of Xinjiang Phytomedicine Resource and Utilization of Ministry of Education, College of Life Sciences, Shihezi University, Shihezi, China; 2 China Colored-cotton (Group) Co., Ltd., Urumqi, China; East Carolina University, UNITED STATES

## Abstract

Chilling stress generates significant inhibition of normal growth and development of cotton plants and lead to severe reduction of fiber quality and yield. Currently, little is known for the molecular mechanism of brown-fiber cotton (BFC) to respond to chilling stress. Herein, RNA-sequencing (RNA-seq)-based comparative analysis of leaves under 4°C treatment in two different-tolerant BFC cultivars, chilling-sensitive (CS) XC20 and chilling-tolerant (CT) Z1612, was performed to investigate the response mechanism. A total of 72650 unigenes were identified with eight commonly used databases. Venn diagram analysis identified 1194 differentially expressed genes (DEGs) with significant up-regulation in all comparison groups. Furthermore, enrichment analyses of COG and KEGG, as well as qRT-PCR validation, indicated that 279 genes were discovered as up-regulated DEGs (UDEGs) with constant significant increased expression in CT cultivar Z1612 groups at the dimensions of both each comparison group and treatment time, locating in the enriched pathways of signal transduction, protein and carbohydrate metabolism, and cell component. Moreover, the comprehensive analyses of gene expression, physiological index and intracellular metabolite detections, and ascorbate antioxidative metabolism measurement validated the functional contributions of these identified candidate genes and pathways to chilling stress. Together, this study for the first time report the candidate key genes and metabolic pathways responding to chilling stress in BFC, and provide the effective reference for understanding the regulatory mechanism of low temperature adaptation in cotton.

## Introduction

Chilling stress results in significant negative effects during each stage of plant life from germination to maturity, leading to destroyed inhibition of plant growth and severe reduction of yield and quality of crops [1]. In higher plants, after exposure to chilling condition, cellular physiological processes containing membrane fluidity, cytoskeletal reorganization, and nucleic acid and protein structures were changed to respond the external stress [2]. The investigations on cold responsive genes and corresponding signaling pathways have been demonstrated properly at the molecular level [3]. In which, the *C-REPEAT BINDING FACTOR* (*CBF*) signaling is the best elucidated pathway in *Arabidopsis* [4]. *CBFs* belong to the members of *ETHYLENE RESPONSE FACTOR/APETALA2* (*ERF/AP2*) family, and are the important regulator to respond to chilling stress through binding to the *cold-responsive* (*COR*) gene promoters and thus inducing their expression levels. *Arabidopsis INDUCER OF CBF EXPRESSION1* (*ICE1*) showed constitutive expression under cold condition [2, 5]. Attempts to decipher the key factors on cold stress by high throughput transcriptomic analysis have been performed in many plants including *A*. *thaliana*, *Glycine max*, *Prunus persica*, and *Mangifera indica* [6–9], identifying large amounts of crucial genes and metabolic pathways. Cell signaling such as the calcium influx from extracellular to cytosol is also reported for plants to adapt chilling stress [10].

To survive under chilling stress, plants evolved comprehensive defense mechanism in which, the adaptive responses at different levels of morphological, physiological, biochemical, and molecular changes were triggered [11–15]. Cold stress could lead to the cell wall destruction by affecting the wall thickness and rigidity, accordingly, the structural changes in cell wall components and significant accumulations of genes and enzymes that mediated cell wall plasticity/rheology were induced for plants to resist the harmful effects [16–18]. A higher lipid content and unsaturation are the positive factors to increase the tolerance to low temperature (LT) in peach by keeping the plasma membrane to be more integrity and mobility [19]. Changes of lipid metabolism including phospholipids and sphingolipids content and constituent were observed to protect the membrane integrity during cold acclimation stage [20, 21]. At the organelle level, chloroplasts, which are responsible for photosynthesis, are the first affected organelles for plants to sense the external LT condition, showing the negative effects on the changes of ultrastructure and photoinhibition [22]. Genetic evidences indicated that the proteins involving in the stability and repair of photosynthesis play important roles in response to chilling stress to maintain the normal structure and function of chloroplasts [23, 24]. Chilling-induced accumulations of intracellular solutes such as proline and total soluble sugars were observed in cold-resistant plants [25]. Besides, phytohormones for instance GA, ABA and BR are important regulators for plants to respond to LT by improving the chlorophyll content and photosynthesis, triggering the downstream signaling pathways, and inducing the expression of genes and proteins associated with cold tolerance [26, 27].

Upland cotton cultivars are important crop plants for their preferential support of the raw fiber materials to the textile industry [28]. For the advantages in eliminating dying costs and avoiding the use of toxic dye, the naturally colored cotton (NCC) varieties were the important members to supply the natural colored fibers, reflecting the increasing demand to utilize the natural green products to benefit the human health and excellent environment [29]. The varieties of brown-fiber cotton (BFC) are the major cultivars for their dominance in fiber quality and yield, and were planted mainly in regions of Xinjiang province in China. However, the low temperature commonly appeared from the end of March to the whole April in Xinjiang and significantly generated inhibition growth or death of the cotton seedlings to lead to a severe reduction of fiber quality and yield. Therefore, it is necessary to elucidate the response mechanism to LT condition and then to breed LT-tolerant NCC cultivars to decrease the negative effect. Current concerns for NCCs were mainly focused on physicochemical characters [30], pigment biosynthesis [31, 32], and quality in colored fibers [32]. To date, there is rare report about the molecular mechanism of NCCs in response to chilling stress. In this study, RNA-sequencing (RNA-seq)-based comparative transcriptome analysis was performed on 4°C-treated leaves of chilling-sensitive (CS) XC20 and chilling-tolerant (CT) Z1612 BFC cultivars. Our results report the candidate key genes and metabolic pathways involving in the response to chilling stress, and provide the possible regulatory mechanism controlling the low temperature adaption in cotton.

## Materials and methods

### Plant material and treatments

The BFC cultivars Z1612 and XC20 were obtained from Xinjiang colored cotton company. The full granule and germinated seeds were cultured on nutrient soil: vermiculite: perlite = 3:1:1 in an automatic climate chamber (200 μmol m^−2^·s^−1^ light intensity, 50–55% relative humidity, 14 h light/10 h dark photoperiod in 28°C culture temperature). Three replicates of 10-day-old seedlings were transferred at 4°C for four days and then placed at 28°C for successive normal growth for seven days. The 10-day-old seedlings of Z1612 and XC20 were treated by 4°C, the treated leaf materials were collected for quick freezing in liquid nitrogen immediately with the storage at −80°C for further use.

### RNA extraction and RNA-seq library construction

Total RNA was extracted from different leaf tissues using RNAprep pure plant kit (TIANGEN, Beijing, China), and was monitored by 1% agarose gels. The RNA purity, concentration, and integrity were checked using a NanoPhotometer^®^ spectrophotometer (IMPLEN, CA, USA), Qubit^®^ RNA Assay Kit and a Qubit^®^ 2.0 Flurometer (Life Technologies, CA, USA), and the RNA Nano 6000 Assay Kit of the Bioanalyzer 2100 system (Agilent Technologies, CA, USA), respectively. Quantified RNA was used to generate sequencing libraries using NEB Next UltraTM RNA Library Prep Kit for Illumina (NEB, CA, USA), and library quality was assessed on the Agilent Bioanalyzer 2100 system (Agilent Technologies, CA, USA). The index-coded samples were clustered by cBot Cluster Generation System using TruSeqPE Cluster Kit v3-cBot-HS (Illumia). The library preparations were generated using an Illumina Hiseq 2500 platform (BMKCloud, Beijing, China). The assembled data was submitted to Sequence Read Archive (SRA) database at National Center for Biotechnology Information Search database (NCBI) with the submission number SUB8146094.

### Transcriptome profiles analysis

cDNA libraries were sequenced using the Sequencing by Synthesis (SBS), clean data were obtained by filtering a large number of raw data and were aligned to the reference genome of *Gossypium hirsutum* (https://phytozome.jgi.doe.gov/pz/portal.html) using TopHat v2.0.12 [33]. Gene function was annotated based on the seven databases, NCBI non-redundant protein sequences (Nr), NCBI non-redundant nucleotide sequences (Nt), Protein family (Pfam), Clusters of Orthologous Groups of proteins (COG), Swiss-Prot, Kyoto Encyclopedia of Genes and Genomes (KEGG) Ortholog database (KO), and Gene Ontology (GO). Gene expression levels were estimated by FPKM [34]. A threshold value of *P* value ≤ 0.05 and log2FoldChange ≥ 1.5 were used to confirm the differential expression analysis. The statistical enrichment of DEGs was tested by KOBAS software in the KEGG database. The cluster of DEGs was analyzed by STEM.

### Validation of RNA-seq data by qRT-PCR

qRT-PCR was performed using the SYBR Premix Ex Taq (Takara, Kusatsu, Japan) and the designed specific primers (S3 Table) on the LightCycler 480 II System (Roche, Basel, Switzerland). The relative expression levels of the target genes were calculated through the 2^-ΔΔCt^ method using the *G*. *hirsutum ubiquitin 7* (*GhUBQ7*) as internal control.

### Determination of physiological and biochemical indexes of BFC cultivars under 4°C treatment

The 0.5 g leaves of BFC cultivars Z1612 and XC20 were ground fully with the addition of 2 mL 6% TCA, with subsequent 8000 rpm centrifugation at 4°C for 15 min. The supernatant was used for the determination of AsA content. The content of total protein was determined by coomassie brilliant blue G-250 method. After addition of distilled water into the homogenate and 3000 rpm centrifugation for 10 min, the supernatant was collected for total protein measurement. The determination of soluble sugar, enzyme activities of APX, SOD and CAT, and contents of proline and MDA were assayed by detection kits (Solarbio, Beijing, China).

## Results

### Phenotype of two BFC cultivars Z1612 and XC20 under 4°C treatment

**Fig 1:**
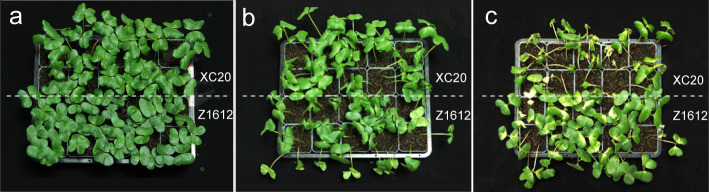
Phenotype of two BFC cultivars Z1612 and XC20. The 10-day-old BFC seedlings under the condition of normal culture (a), 4-day 4°C treatment (b), and 7-day recovery from 4°C treatment (c) were photographed for phenotype analysis.

The 10-day-old seedlings of the two BFC cultivars Z1612 and XC20 were treated by 4°C for four days and then placed at 28°C for successive normal growth for seven days, and the generated plants were used for phenotype analysis. The results showed that, lots of brown frozen spots were observed on cotyledon of XC20 and little appeared on cotyledon of Z1612 (Fig 1). Meantime, the statistical analysis of death rate (DR) of seedlings indicated that, a high DR in XC20 and low DR in Z1612 were obtained (S1 Fig). These data demonstrated that, Z1612 and XC20 could be identified as chilling-tolerant (CT) and chilling-sensitive (CS) cultivars respectively. The leaves of these two cultivars treated by 4°C for different time were collected for RNA-Seq analysis.

### Analysis of Differentially Expressed Genes (DEGs) in two BFC cultivars under 4°C treatment

**Fig 2:**
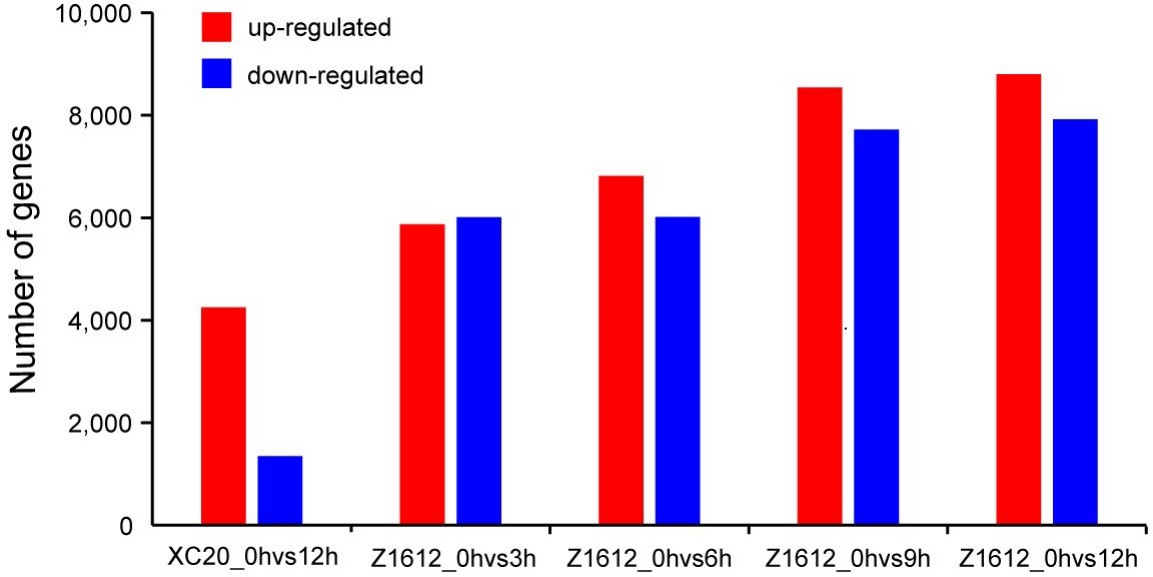
Statistics analysis of DEGs in two BFC cultivars of CT Z1612 and CS XC20 under 4°C treatment. The DEGs were identified with the filter conditions of FPKM Log2 fold change ≥ 1.5 and adjusted *p* value ≤ 0.05 between each comparison group. Red and blue columns represent up-regulated and down-regulated DEGs respectively.

To identify genes correlated with the response to chilling stress, RNA-seq-based transcriptome analysis of the cotyledon of two BCF cultivars Z1612 and XC20 that appeared different chilling tolerance was performed. The cotton plants were treated by 4°C with different times (3, 6, 9, and 12 h), and the resulted cotyledon materials were extracted for RNA isolation that was then used for sequencing with three biological replicates. Box line diagram analysis indicated that the transcriptome datasets were high quality and preferable sensitivity (S2 Fig). By *de novo* assemble of the generated 150.54 Gb clean data with the average clean data of 5.89 Gb for each sample and the Q30 value of 92.21% (S1 Table), a total of 72650 unigenes were annotated by subjecting them to the eight commonly used functional databases (S2 Table). The unigenes annotated by COG and KEGG databases were used for subsequent analysis of function prediction and classification. To better understand the difference between the CT Z1612 and CS XC20 responding to chilling stress, differentially expressed genes (DEGs) were identified with the filter conditions of fragments per kilo base of transcript sequence per millions base pairs sequenced (FPKM) Log2 fold change ≥ 1.5 and adjusted *P* value ange ≥ 1.5 and adjusted ence between the CT Z1612 and CS XC20 4251, 5874, 6818, 8539, and 8802 genes were identified as up-regulated DEGs (UDEGs) in the comparison groups XC20_0hvs12h, Z1612_0hvs3h, Z1612_0hvs6h, Z1612_0hvs9h and Z1612_0hvs12h, respectively (Fig 2).

### Analysis of the Up-regulated DEGs (UDEGs) in different comparison groups

**Fig 3:**
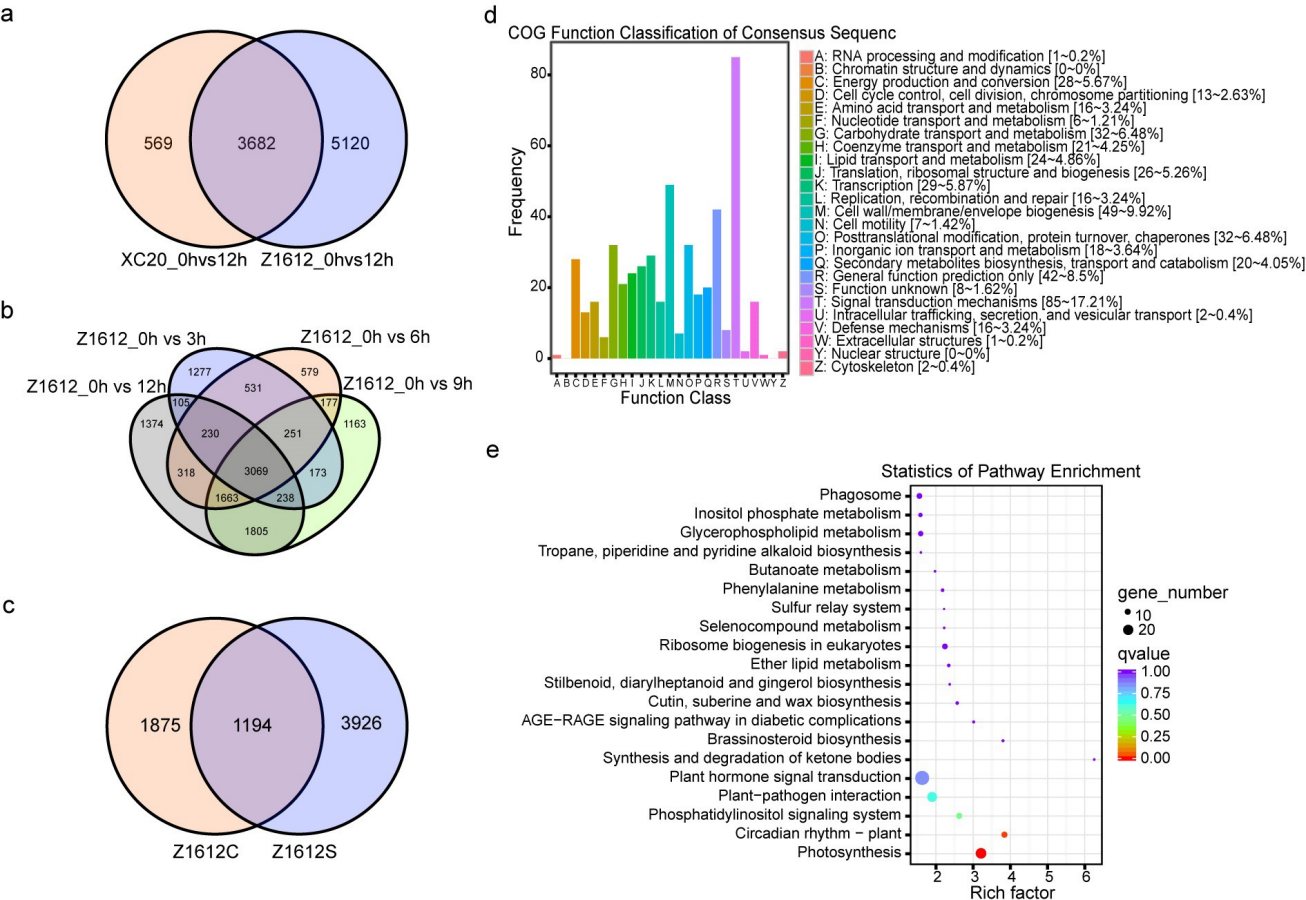
Analysis of the UDEGs in different comparison groups. a: Venn diagram analysis of the UDEGs in XC20_0hvs12 hand Z1612_0hvs12h, resulting 5120 specific-expressed UDEGs (Z1612S). b: Venn diagram analysis of the UDEGs in different comparison groups of Z1612_0hvs3h, Z1612_0hvs6h, Z1612_0hvs9h, and Z1612_0hvs12h, generating 3069 co-expressed UDEGs (Z1612C). c: Venn diagram analysis of UDEGs in Z1612S and Z1612C, discovering 1194 joint-expressed UDEGs (Z1612SC). COG (d) and KEGG (e) analyses of the 1194 UDEGs of Z1612SC, different color columns represent different function classifications.

To investigate genes possibly participating in the response to chilling stress, the UDEGs of each comparison group, especially in the comparison groups of Z1612, were analyzed by venn diagram (Fig 3A–3C). The results indicated that 3682 UDEGs were co-expressed in the comparison groups of XC20_0hvs12h and Z1612_0hvs12h, and that 5120 UDEGs (Z1612S) were specifically expressed in the group of Z1612_0hvs12h (Fig 3A). To further identify genes potentially regulating chilling response in the CT cultivar Z1612, the up-regulated genes in each comparison group of Z1612_0hvs3h, Z1612_0hvs6h, Z1612_0hvs9h and Z1612_0hvs12h, were analyzed by Venn diagram, showing 3069 UDEGs (Z1612C) were co-expressed in the four groups (Fig 3B). Successive joint analysis of the co-expressed and specific-expressed genes in groups of Z1612C and Z1612S displayed that 1194 UDEGs (Z1612SC) were identified (Fig 3C). Subsequently, the 1194 UDEGs of Z1612SC were subjected to COG and KEGG to discover the gene distribution pathways. COG analysis showed that signal transduction mechanisms (85~17.21%), cell wall/membrane/envelope biogenesis (49~9.92%), general function prediction only (42~8.5%), posttranslational modification, protein turnover, chaperones (32~6.48%), and carbohydrate transport and metabolism (32~6.48%), were the major enriched terms (Fig 3D). KEGG analysis indicated that photosynthesis, circadian rhythm-plant, phosphatidylinositol signaling system, plant-pathogen interaction, and plant hormone signal transduction were the significant enriched pathways (Fig 3E). These results indicate that, the UDEGs involved in signal transduction, phytosynthesis, and protein and carbohydrate related metabolisms may be important for the response to chilling stress.

### Analysis of UDEGs with constant increased expression in different comparison groups of Z1612

**Fig 4:**
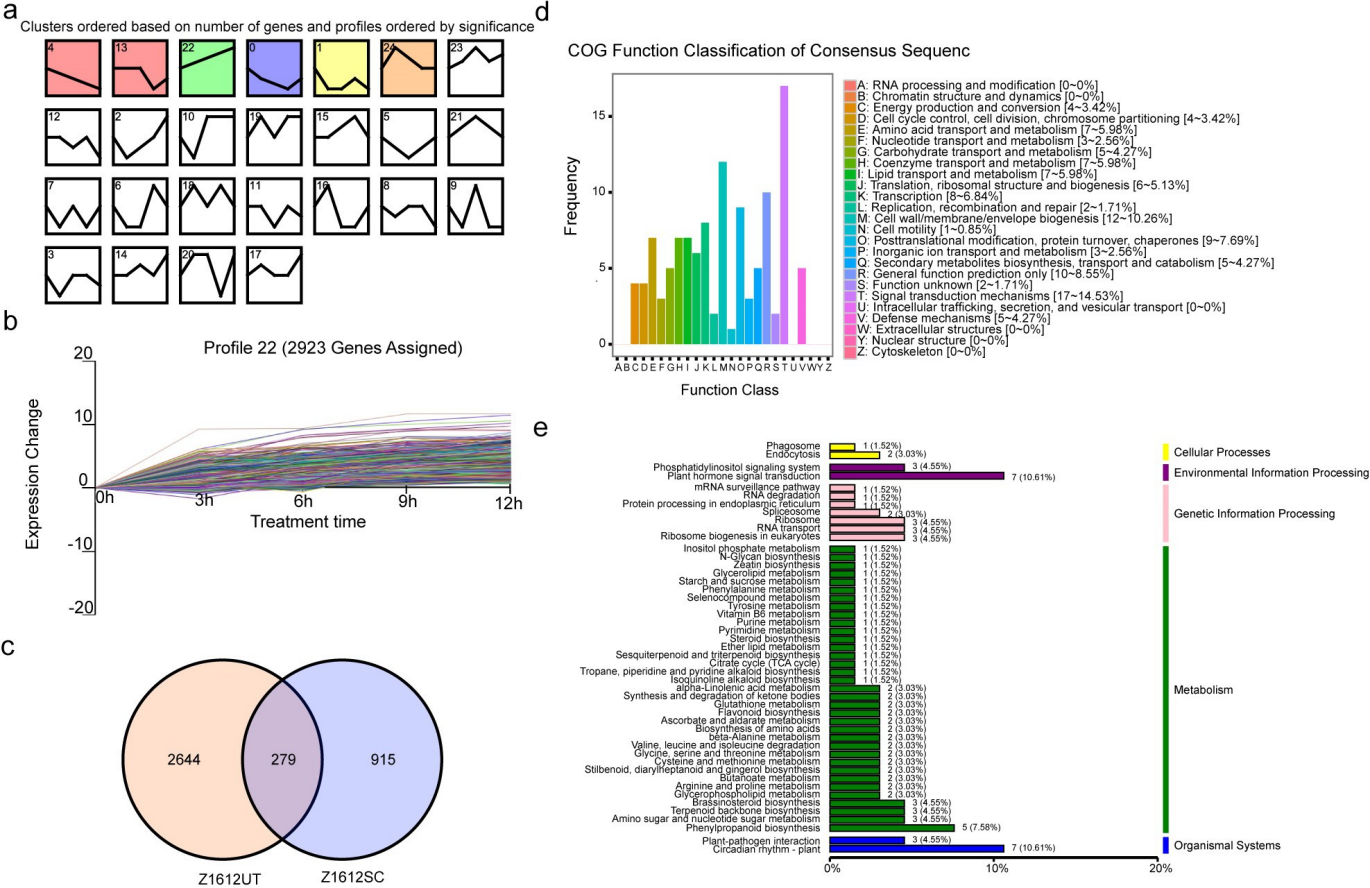
Analysis of UDEGs with constant increased expression in Z1612 groups. a: The 25 classified clusters were obtained according to the expression profiles of the UDEGs of Z1612 groups by K-means algorithm STEM software. The colored clusters denote the profiles that have the value of *p* < 0.001. b: Expression profiles of the 2923 UDEGs (Z1612UT) of cluster 22 appearing constant increased expression levels during different stages of 4°C treatment. c: Venn diagram analysis of UDEGs in Z1612UT and Z1612SC, identifying 279 UDEGs in both datasets. COG (d) and KEGG (e) analyses of the 279 UDEGs, different color columns indicate different function classifications.

Since the expression difference might be existed in the UDEGs in different comparison groups of Z1612, to excavate the UDEGs with constant increased expression along with the sequential time increase of 4°C treatment in Z1612, by K-means algorithm Short Time-series Expression Miner (STEM) software, with 25 being identified (Fig 4A). In which, the cluster 22 showed the strongest connection with the profile of persistent high expressions along with the continuous chilling treatment (Fig 4B). A total of 2923 UDEGs (Z1612UT) were included in cluster 22 and were then compared with the UDEGs of Z1612SC that showed increased expressions in all comparison groups, resulting in the identification of 279 UDEGs that were present in both datasets (Fig 4C). Therefore, these genes are UDEGs in all comparison groups of Z1612 with constant high abundance at the dimensions of both each comparison group and treatment time, and may be the potential important candidate genes that are crucial for chilling stress response in the process of continuous 4°C treatment. The 279 UDEGs were further analyzed by COG and KEGG. The COG result showed that signal transduction mechanisms (17~14.53%), cell wall/membrane/envelope biogenesis (12~10.25%), and general function prediction only (10~8.55%) were the major enriched terms (Fig 4D). The KEGG analysis indicated that, circadian rhythm-plant (7~10.45%) and plant-pathogen interaction (3~4.48%) in organism systems; plant hormone signal transduction (7~10.45%) and phosphatidylinositol signaling system (3~4.48%) in environmental information processing; ribosome biogenesis in eukaryotes (3~4.48%), RNA transport (3~4.48%) and ribosome (3~4.48%) in genetic information processing; and the pathways related to metabolism such as phenylpropanoid biosynthesis (5~7.46%), amino sugar and nucleotide sugar metabolism (3~4.48%), brassinosteroid biosynthesis (3~4.48%), and terpenoid backbone biosynthesis (3~4.48%), etc, were discovered as significant enriched pathways (Fig 4E). Meanwhile, to validate the accuracy of the RNA-seq experiment, fifteen out of the 279 UDEGs were selected for real-time quantitative polymerase chain reaction (qRT-PCR) detection. The qRT-PCR results indicate a high consistency with the RNA-seq data (S3 Fig). These results sustain the hypothesis again that the UDEGs related to signal transduction, protein and carbohydrate metabolism, and cell component may contribute critical role for chilling response.

### Physiological index and cellular metabolic substance detection

**Fig 5:**
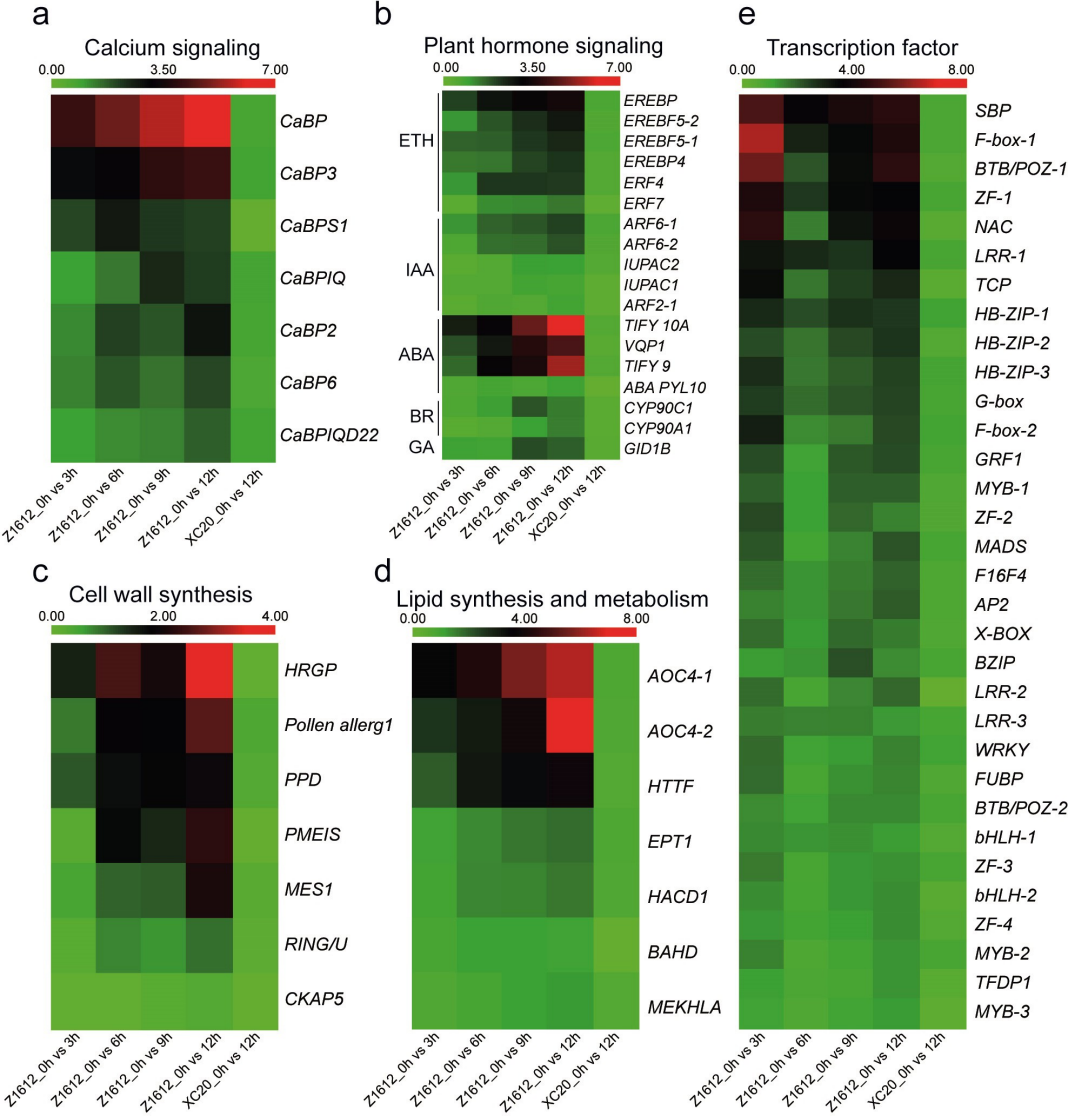
Expression analysis of the UDEGs involving in signaling pathway. Transcriptome heatmap representing the UDEGs related to calcium signaling (a), plant hormone signaling (b), cell wall synthesis (c), lipid synthesis and metabolism (d), and transcription factors (e), respectively. The transcript levels were indicated by different colors with red and green to represent high and low expressions, respectively. The visualized heatmap was generated using R package on the basis of the FPKM values.

The increase of freezing tolerance in plants is due to reprogramming of gene expression which results in multiple levels of biochemical and cell biological changes [33–36]. Therefore, we further analyzed the expression levels of the 279 UDEGs that located in different pathways, and detected the physiological indexes and cell metabolic substances, so as to reveal the response mechanism to cold stress. Ca^2+^ is an important second messenger to change the cytosolic concentration in response to environmental and developmental stimuli [10]. Seven significant increased UDEGs were classified into calcium signaling pathway, of which *Ca*^*2+*^*-binding protein 1* (*CaBP*) and *calcium-binding EF-hand family protein* (*CaBP3*) appeared the most significant enrichment in Z1612 (Fig 5A). Since “Plant hormone signal transduction” was the enriched KEGG term, we analyzed the expression patterns of UDEGs distributing in this pathway. The UDEGs in plant hormone signaling pathway were involved in the signal transduction of ethylene (ETH), indole-3-acetic acid (IAA), abscisic acid (ABA), brassinolide (BR), and gibberellin (GA), and most UDEGs in Z1612 indicated a rapid increase under chilling stress with the peak values after 12 h treatment. Interestingly, the UDEGs in ABA pathway indicated the highest expression level than that in ETH, IAA, BR, and GA pathways, maintaining a constant increased level from 3 to 12 h treatment, implying the important positive role of ABA to chilling stress (Fig 5B). Additionally, expression of cell wall synthesis related genes including *hydroxyproline-rich glycoprotein family protein* (*HRGP*), *pollen allerg1*, *pheophorbidase* (*PPD*), *pectin methylesterase inhibitor superfamily protein* (*PMEIS*), and *methyl esterase 1* (*MES1*) were significantly up-regulated in Z1612 (Fig 5E). Expression of several lipid synthesis and metabolism genes, containing *Allene-oxide cyclase 4–1* (*AOC4-1*), *AOC4-2*, and *AAA-type ATPase like protein* (*HTTF*) were accumulated in Z1612 under 4°C treatment (Fig 5D). Thirty two out of the 279 UDEGs were identified as transcription factors (TFs), in which *squamosa promoter-binding protein-like* (*SBP domain*) *transcription factor family protein* (*SBP*), *F-box family protein 1* (*F-box-1*), *BTB and TAZ domain protein 1* (*BTB/POZ-1*), *zinc finger protein-like 1* (*ZF-1*), *NAC*, and *leucine rich repeat 1* (*LRR-1*) showed prompt enrichment after 3 h treatment, suggesting their potential important role in regulating downstream gene expression (Fig 5E). Regarding the low expression of the above UDEGs in CS cultivar XC20, these results indicated that the enriched UDEGs locating in signaling pathways may play important function for CR cultivar Z1612 to respond to chilling stress.

**Fig 6:**
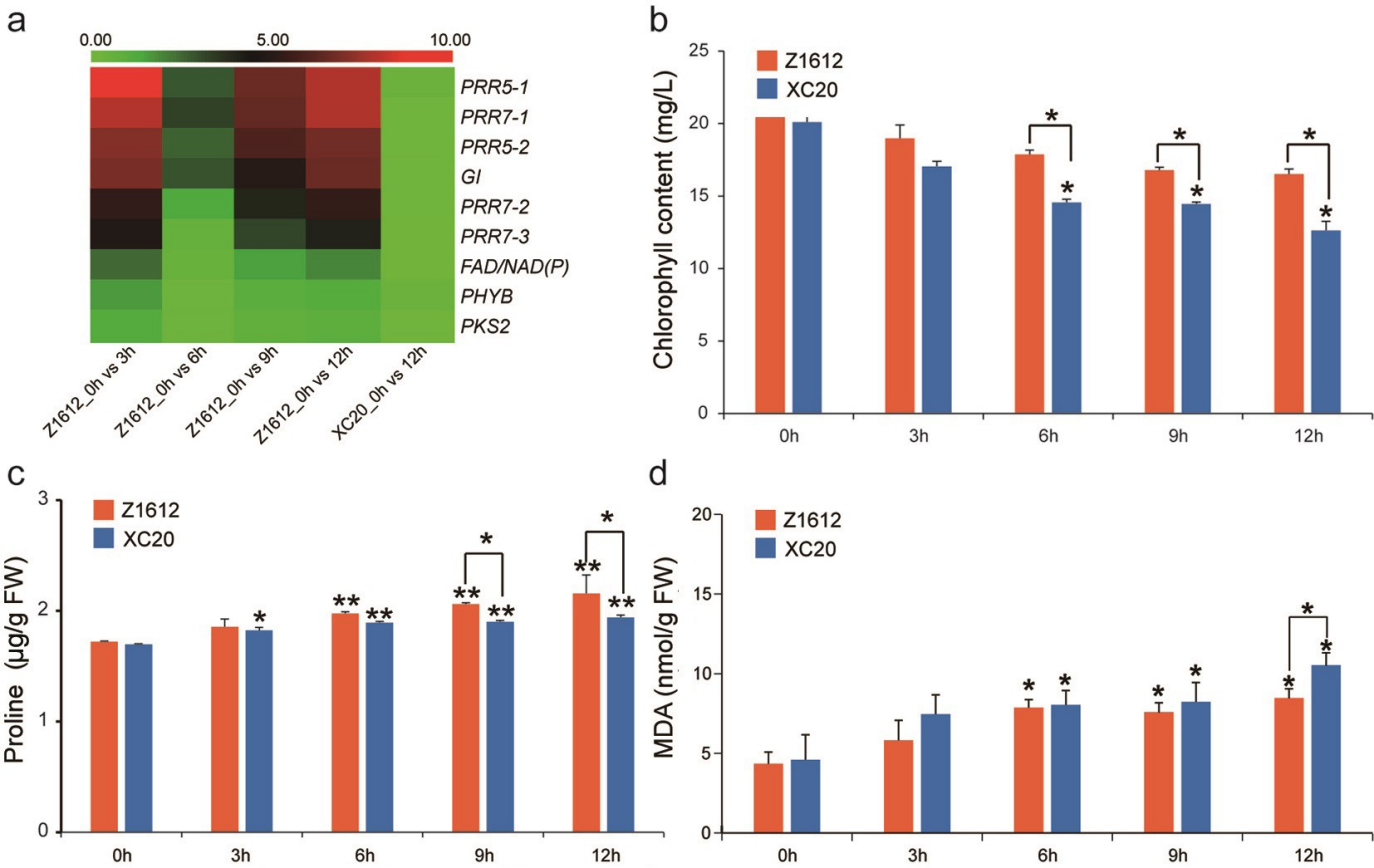
Expression analysis of the UDEGs related to photosynthesis and detection of chlorophyll, proline, and MDA content under 4°C treatment. a: Transcriptome heatmap representing the UDEGs related to photosynthesis. Different colors represent diverse expression levels with red and green to indicate high and low abundances respectively. The visualized heatmap was produced by R package based on FPKM values. Detections of chlorophyll (b), proline (c), and MDA (d) contents. Each value represents the average the three independent experiments. Statistical analysis was performed by independent samples *t*-test, with * and ** to denote the difference at 0.05 and 0.01 level respectively.

Chloroplasts are highly sensitive to chilling stress, which alters the concentration of chlorophyll to adapt to low-temperature conditions [22]. To investigate the changes of chloroplast, we analyzed the expression of the chloroplast related UDEGs and measured the content of chlorophyll and cellular substance of proline and malondialdehyde (MDA). The results indicated that, the photosynthesis related UDEGs including *pseudo-response regulator 5 (PRR5-1* and *PRR5-*2), *pseudo-response regulator 7* (*PRR7-1*/*2*/*3*), *gigantea protein* (*GI*), and *FAD/NAD(P)-binding oxidoreductase family protein* (*FAD/NAD(P)*), were significantly up-regulated in 3-, 9- and 12 h-treated Z1612 materials (Fig 6A). The total chlorophyll content showed no obvious change in Z1612 and significant decrease after 6 h treatment in XC20, with the significant difference between Z1612 and XC20 from 6 to 12 h treatment also being observed (Fig 6B). The proline content of Z1612 and XC20 both showed an increased tendency after 4°C treatment, and significant higher accumulations in 9- and 12 h-treated Z1612 were detected compared with the corresponding XC20 (Fig 6C). Meantime, the MDA content presented gradual ascend in Z1612 and XC20, but held significant lower level in Z1612 than that in XC20 after 12 h 4°C treatment (Fig 6D). These results suggest that higher chlorophyll content and better photosynthesis maintaince, more proline accumulation and less MDA generation provide the preferable ability to resist chilling stress.

**Fig 7:**
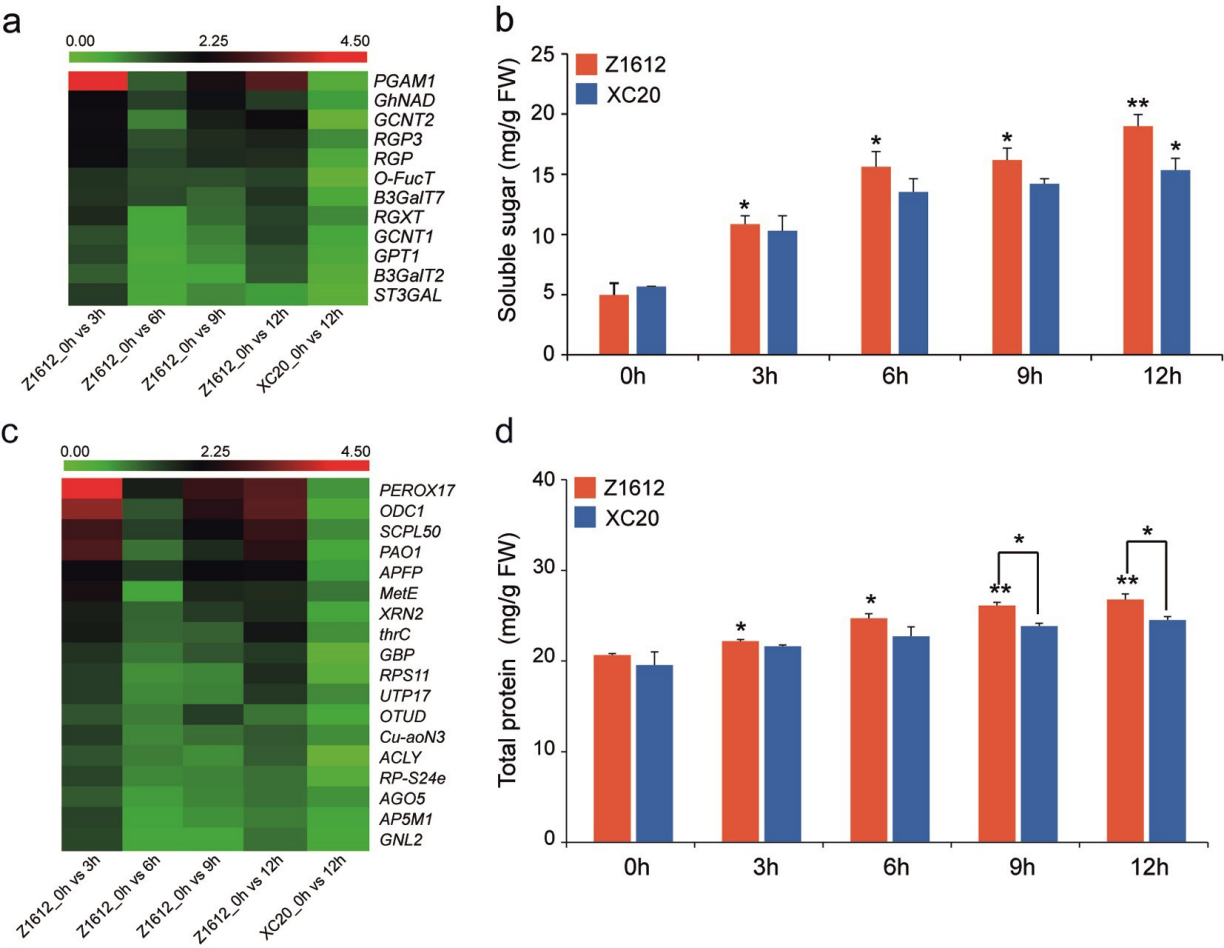
Expression analysis of sugar and protein metabolism related UDEGs and measurement of soluble sugar and total protein under 4°C treatment. Heatmap of expression levels of sugar (a) and protein (c) metabolism related UDEGs. Different colors indicate the diverse expression levels with red and green to present high and low abundances, respectively. The visualization of heatmap was generated by R package according to the obtained FPKM values. Measurement of soluble sugar (b) and total protein (d). Each value represents the average of three independent experiments with observance of mean value±SD. Statistical analysis of significant difference was performed by one-way ANOVA, with * and ** to denote the difference at 0.05 and 0.01 level respectively.

Considering sugars and proteins as important factors to contribute to the stabilization of membrane phospholipids, thereby to protect the membranes against freeze damage [19, 25], we analyzed the transcript expression of sugar and protein related genes in the 279 UDEGs, and tested the content of soluble sugar and protein in CT Z1612 and CS XC20 cultivars under 4°C treatment. The results showed that, sucrose-metabolism related UDEGs were accumulated in Z1612 under 4°C treatment, especially *phosphoglycerate mutase-like protein 1* (*PGAM1*) indicated the significant highest expression level in 3 h-treated Z1612 (Fig 7A). Meanwhile, the soluble sugar content of Z1612 appeared significant rapid increase after 3 h treatment and maintained constant elevation thereafter, while XC20 indicated relative slow raise without difference till the treatment time of 12 h (Fig 7B). For the protein related UDEGs, *peroxidase superfamily protein 17* (*PEROX 17*), *ornithine decarboxylase 1* (*ODC1*), *serine carboxypeptidase-like 50* (*SCPL50*), and *polyamine oxidase 1* (*PAO1*) showed the highest enrichments promptly in 3 h-treated Z1612, and held a relative abundant expression at the treatment time of 9 and 12 h (Fig 7C). The total protein content of Z1612 displayed significant increase after 3 h treatment, and appeared significant difference in 9 and 12 h-treated Z1612 compared to that in XC20 that showed relative steady range without obvious change (Fig 7D). On the basis of these data, it is suggested that the significant enriched intracellular substances of soluble sugar and total protein may play important role in maintaining the integration of cell structure and regular energy metabolism under chilling stress.

**Fig 8:**
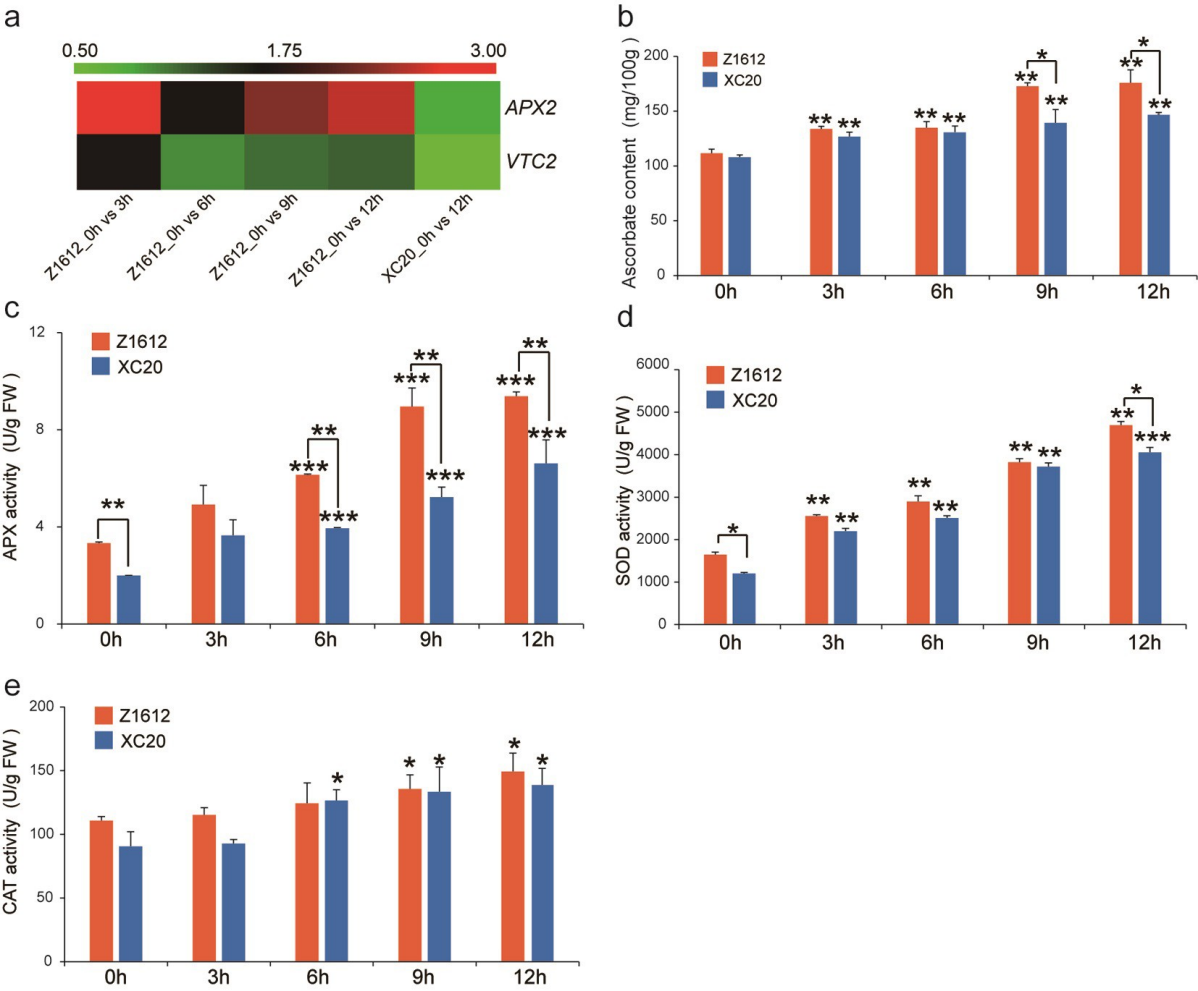
Analysis of AsA antioxidative system metabolism. a: Transcriptome heatmap of expression levels of the UDEGs related to ascorbate metabolism. The expression levels of *APX2* and *VTC2* were showed by different colors with red and green to indicate high and low abundances, respectively. The heatmap was produced by R package according to the FPKM values of the transcriptome data. b: Detection of ascorbate content. Measurement of enzyme activities of APX (c), SOD (d), and CAT (e). The values were indicated with mean±SD by three independent experiments. Statistical analysis of significant difference was performed by independent samples *t*-test, *, **, and *** represent *p* < 0.05, *p* < 0.01, and *p* < 0.001, respectively.

Under chilling stress, reactive oxygen species (ROS)-scavenging enzymes are activated, and antioxidants also are accumulated, to mitigate the increased ROS and thus to maintain the cellular normal homeostasis [34]. The transcriptome data indicated that, *ascorbate peroxidase 2* (*APX2*) gene showed a significant high abundance, and a ascorbic acid (AsA) *de novo* biosynthesis gene *VTC2* displayed increased expression, in the CT cultivar Z1612 under 4°C treatment (Fig 8A), implying the possible important function of AsA synthesis and AsA-mediated antioxidative metabolism in LT adaptation. The AsA content of both Z1612 and XC20 indicated a prompt elevation after 3 h treatment of 4°C, and Z1612 showed more significant AsA accumulation than in XC20 under 9 and 12 h treatment (Fig 8B). For the antioxidative enzymes, APX, SOD, and CAT appeared gradual increase in both Z1612 and XC20 (Fig 8C–8E). Compared to XC20, Z1612 had significant higher activities of APX at initial point (0 h) and 6, 9, and 12 h treatment time, and of SOD at 0 and 12 h stimulation (Fig 8C and 8D), without obvious difference for the CAT activity (Fig 8E). These results suggest that AsA and its mediated antioxidative enzymes may be the important regulator for cotton to resist chilling stress by controlling the cellular redox homeostasis.

## Discussion

### Analysis of the DEGs in chilling-tolerant cultivar Z1612

BFC varieties are the major cultivated NCCs. By screening the BFC varieties under 4°C treatment, we identified the Z1612 as the CT cultivar and XC20 as the CS cultivar respectively (Fig 1). The obtained two different chilling-tolerant BFC cultivars were then treated under 4°C for 0–12 h for successive RNA-seq-based transcriptome analysis, to identify the candidate key genes and metabolic pathways involving in chilling stress response. Transcriptomic approach has been widely utilized to investigate the molecular in response to chilling stress in several plants, such as *populus simonii*, *sorghums*, and rice [3, 37, 38]. The previous transcriptome analysis in BFC has focused mainly on fiber quality and color [32, 39], but little is known about chilling stress responsive. Our study for the first time analyzed the different tolerance for BFC cultivars and performed the comparative transcriptome analysis of the two typical different chilling-tolerant cultivars.

Many genes and pathways are involved in plant response to cold stress [40]. A total of 8802 UDEGs in Z1612_0hvs12h and 4251 in XC20_0hvs12h were identified (Fig 2), indicating more UDEGs that may be crucial for the chilling stress response were found in Z1612, which is consist with the reported results that more UDEGs are commonly discovered in tolerant plants [37]. Under the conditions of fold change ≥ 1.5 and *P* value < 0.05, comprehensive comparative analysis finally obtained 279 UDEGs as the potential most important candidates with the distribution in the pathways of signal transduction, protein and carbohydrate metabolism, and cell component (Fig 4).

### Signal transduction regulation in the response process of chilling stress

Regulation of plant adaptation is often highly complex and signal transduction, including phytohormones, sugar-signaling, and Ca^2+^ are involved in this complexity [25, 41, 42]. Plant hormones such as abscisic acid (ABA) and jasmonic acid (JA) were validated to regulate plant responses [42]. Our data indicated that ABA biosynthesis-related genes (*TIFY10A*, *TIFY9*, and *VQP1*) appeared the most significant up-regulated expressions during 4°C treatment in CT cultivar (Fig 5), which may promote the synthesis of endogenous ABA, showing the consistency with the previous studies that low temperature induces an immediate increase in endogenous ABA levels [35], after which the stomata close to decrease the rate of photosynthesis [12]. ABA might affect the photosynthesis that is one of the key responses to respond to chilling stress [36].

Sugar-signaling is involved in various abiotic stresses [43], and plants commonly change sugar status to modulate sugar metabolism in response to cold stress [25]. The content of soluble sugar and the expression levels of sugar metabolism related UDEGs were significantly increased in CT cultivar Z1612 after chilling stress (Fig 7), which is similar with the investigation that the genes involving in sugar metabolism are up-regulated in response to low temperature [44]. PGAM1 showed the most significant accumulation after 3 and 12 h 4°C treatment (Fig 7), suggesting its required role in promoting repair of damaged DNA molecules [45].

As the ubiquitous second messenger, calcium ion (Ca^2+^) mediates stimuli-response coupling in the regulation of physiological process and chilling stress [10, 41]. Ca^2+^-binding protein family genes, *CaBP* and *CaBP3* transcription levels showed significant enrichment in CT cultivar Z1612 after chilling stress (Fig 5). Ca^2+^ signal may function as a crosstalk factor in response to chilling stress and plant phytoremediation [41]. *Arabidopsis* Ca^2+^-binding protein gene *PCaP2* is highly induced under chilling stress, and is a positive regulator of ABA signaling pathway [46]. In this study, the ABA biosynthesis-related genes indicated significant up-regulation under chilling stress (Fig 5), suggesting the potential regulatory mechanisn that Ca^2+^ and Ca^2+^-binding proteins might associate with ABA to protect integrity of the cells under low temperature by activating *CBF*-meditated transcriptional regulatory pathways [46].

### Effect of chilling stress on chloroplasts

Chilling stress is an adverse environmental signal that alters changes in the redox state of photosynthesis components [47, 48]. Phytochrome B (phyB) level could be enhanced by increased *PHYB* expression and then altered the ability of plants to respond to light signals [49]. Some photosynthesis related UDEGs including *PHYB* and *GI* indicated up-regulated expressions in CT cultivar Z1612 (Fig 6), implying their possible important role in phyB-signal transduction [50]. *PRR7* (*pseudo-response regulator 7*) and *PRR9* (*pseudo-response regulator 9*) are critical elements of a temperature-sensitive circadian system for the *Arabidopsis* clock and are partially functional redundancy [51]. Three *PRR5* and two *PRR7* genes in CT cultivar Z1612 showed significant accumulations after 3, 9, and 12 h chilling stress, suggesting their key role in low temperature responsiveness. Activities of the photosynthesis system are reduced under chilling stress [1]. The content of chlorophyll in CT cultivar Z1612 was higher than that in CS cultivar XC20 (Fig 6B), indicating the chilling-tolerant cotton plants could alleviate the chloroplast damage and thus guarantee the photosynthesis reaction.

### Cytophysiological changes caused by chilling stress

Cell permeability and multiple disorganizations are altered by cell membrane phase transition induced by chilling stress [52]. Glycerophospholipid metabolism is an important pathway that was significantly activated during chilling stress in Z1612 (Fig 4), and is used in the synthesis of phosphatidic acid (PA), an important components of cell membranes in plants [53]. As a key member of cell wall synthesis genes, *HRGP* showed significantly up-regulated expression after chilling stress in Z1612 (Fig 5), indicating the key function of the encoded HRGP protein as the major structural proteins of cell walls, and as the forms of covalent or non-covalent linkages to other HRGPs, to enhance the wall under stress [54]. Two *AOC4* genes were key members of lipid synthesis and metabolism with high expressions after chilling stress in Z1612 (Fig 5). *AOCs* involve in JA biosynthesis that is the key regulator upstream of cold-regulated TFs, such as *CBF* [55, 56]. The major organic osmolyte of proline was accumulated under chilling stress (Fig 6), showing its function to influence enzyme and membrane integrity along with positive effects under stress conditions [57]. MDA is the primary and secondary lipid derivative [23]. MDA levels indicated a lower increase in CT Z1612 than in CS XC20 (Fig 6), which is similar with the studies that, lower MDA content imply more resistance of the plants to chilling stress [58].

Membrane damage could lead to the production of ROS as both signal molecules and toxic byproducts in plant cells [54]. The ROS-scavenging enzymes of APX, SOD, and CAT are activated to guard cells against the oxidative burst [59], our data showed similar results that the expression of antioxidant AsA metabolism genes of *APX2* and *VTC2*, AsA content, and the activities of AsA-mediated antioxidative enzymes of APX, SOD, and CAT, were significantly increased in CT cultivar Z1612 (Fig 8). *APX2* encodes a cytosolic ascorbate peroxidase to protect sites of primary photosynthesis from ROS [60], transgenic plants overexpressing *APX2* enhanced plant chilling stress tolerance [61], by maintaining the intracellular H_2_O_2_ homeostasis against low temperature stress. ROS signals can activate the expression of TFs to exert the effects [2, 62]. TFs of *SBP*, F-*box*-1, and *BTB*/*POZ*-1 appeared higher accumulations in Z1612 after chilling stress (Fig 5), implying their potential role in low temperature adaptation, with further investigation of regulatory mechanism to be expected.

## Supporting information

S1 Fig Statistical analysis of mortality of Brown-Fiber Cotton (BFC) seedlings. The 10-day-old seedlings of 12 BFC cultivars were treated at 4°C for 4 days and then recovered at 28°C for 7 days. The obtained seedlings were used for mortality statistics analysis.
(DOC)

S2 Fig Box line diagram analysis of the gene expression distribution of the 21 test samples. (DOCX)

S3 Fig Correlation analysis of the RNA-seq data and qRT-PCR assay. qRT-PCR detection of the selected 15 out of the 279 UDEGs was performed using the presented materials that were consistent with that for RNA-seq with three independent experiments. FPKM value was obtained from the RNA-seq data. Relative expression was determined by qRT-PCR, and was normalized using *GhUBQ* gene as internal control. The 0 h value was artificially set to 1, and the qRT-PCR-based heatmap was generated by R package. Gene numbers were shown in the middle of the diagram and the corresponding primers for qRT-PCR were listed in S3 Table.
(DOCX)

S1 Table Length distribution of all unigenes. (DOCX)

S2 Table Details of functional annotation of all unigenes. (DOCX)

S3 Table Primers used in this work. (DOCX)

## Abbreviations

ABA: Abscisic acid
AsA: Ascorbic acid
BFC: Brown-fiber cotton
BR: Brassinolide
COG: Clusters of Orthologous Groups of proteins
CS: Chilling-sensitive
CT: Chilling-tolerant
DEG: Differentially expressed genes
DR: Death rate
ETH: Ethylene
FPKM: Fragments per kilo base of transcript sequence per millions base pairs sequenced
GA: Gibberellin
IAA: Indole-3-acetic acid
JA: Jasmonic acid
KEGG: Kyoto Encyclopedia of Genes and Genomes
LT: Low temperature
MDA: Malondialdehyde
NCC: Naturally colored cotton
qRT-PCR: Real-time quantitative polymerase chain reaction
ROS: Reactive oxygen species
RNA-seq: RNA-sequencing
STEM: Short Time-series Expression Miner
UDEG: Up-regulated DEG
