# Supplementary material for: Comparative transcriptome analysis of leaves during early stages of chilling stress in two different chilling-tolerant brown-fiber cotton cultivars
Source: PLoS One. 2021 Feb 9;16(2):e0246801. doi: 10.1371/journal.pone.0246801 (PMC7872267; doi:10.1371/journal.pone.0246801)
Supplement: S1 Fig — The 10-day-old seedlings of 12 BFC cultivars were treated at 4°C for 4 days and then recovered at 28°C for 7 days. The obtained seedlings were used for mortality statistics analysis. (DOC) [file pone.0246801.s001.doc]

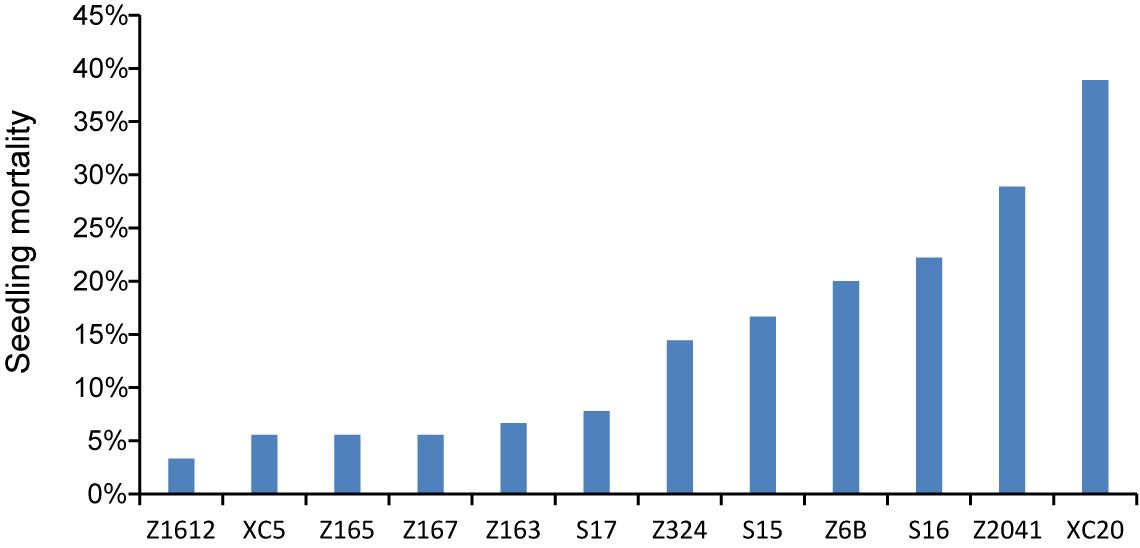


**S1 Fig. Statistical analysis of mortality of brown-fiber cotton (BFC) seedlings.** The 10-day-old seedlings of 12 BFC cultivars were treated at 4℃ for 4 days and then recovered at 28℃ for 7 days. The obtained seedlings were used for mortality statistics analysis.
