# Supplementary figures and images for: Comparative transcriptome analysis of leaves during early stages of chilling stress in two different chilling-tolerant brown-fiber cotton cultivars
Source: PLoS One. 2021 Feb 9;16(2):e0246801. doi: 10.1371/journal.pone.0246801 (PMC7872267; doi:10.1371/journal.pone.0246801)

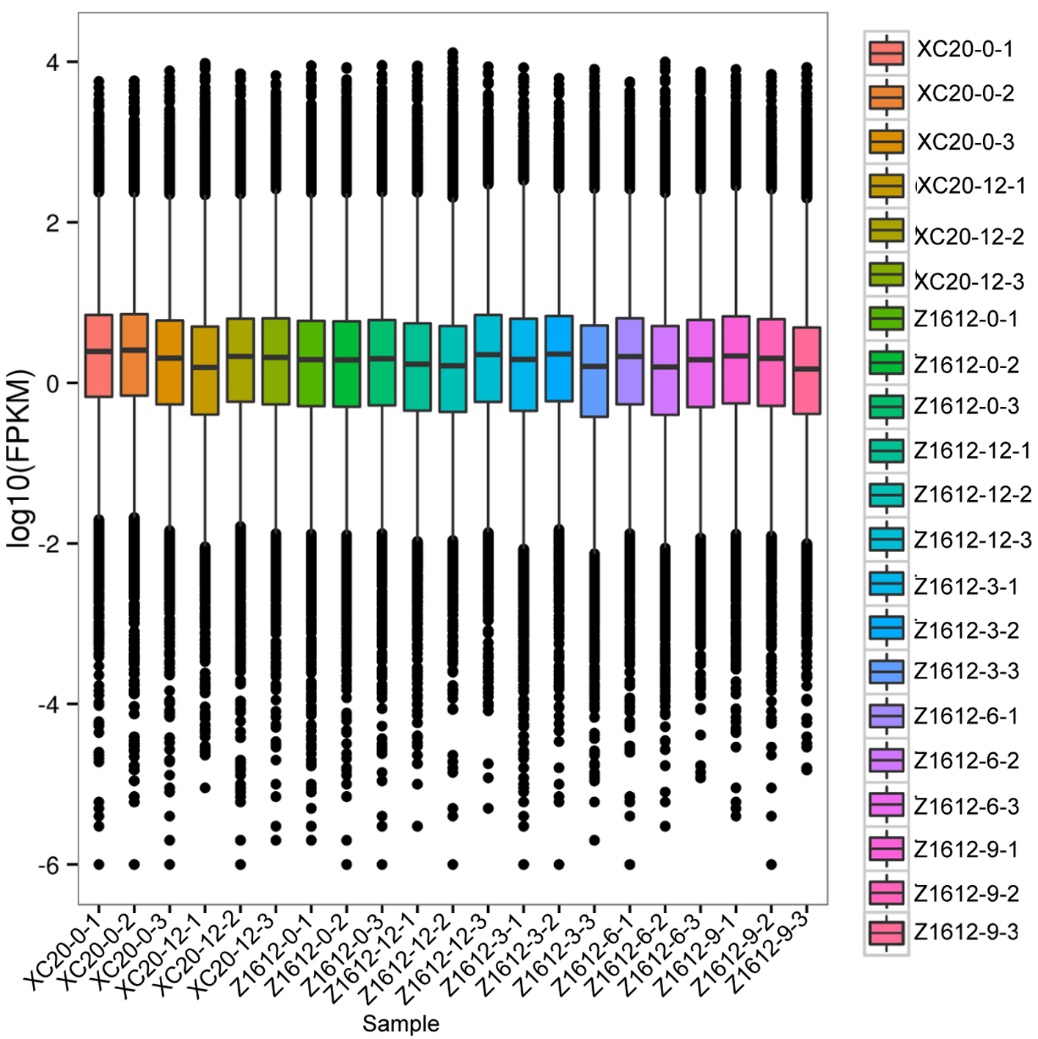


**S2 Fig. Box line diagram analysis of the gene expression distribution of the 21 test samples.**

Supplement: S2 Fig — (DOCX) [file pone.0246801.s002.docx]
