# Supplementary material for: Comparative transcriptome analysis of leaves during early stages of chilling stress in two different chilling-tolerant brown-fiber cotton cultivars
Source: PLoS One. 2021 Feb 9;16(2):e0246801. doi: 10.1371/journal.pone.0246801 (PMC7872267; doi:10.1371/journal.pone.0246801)
Supplement: S3 Fig — qRT-PCR detection of the selected 15 out of the 279 UDEGs was performed using the presented materials that were consistent with that for RNA-seq with three independent experiments. FPKM value was obtained from the RNA-seq data. Relative expression was determined by qRT-PCR, and was normalized using GhUBQ gene as internal control. The 0 h value was artificially set to 1, and the qRT-PCR-based heatmap was generated by R package. Gene numbers were shown in the middle of the diagram and the corresponding primers for qRT-PCR were listed in S3 Table. (DOCX) [file pone.0246801.s003.docx]

**
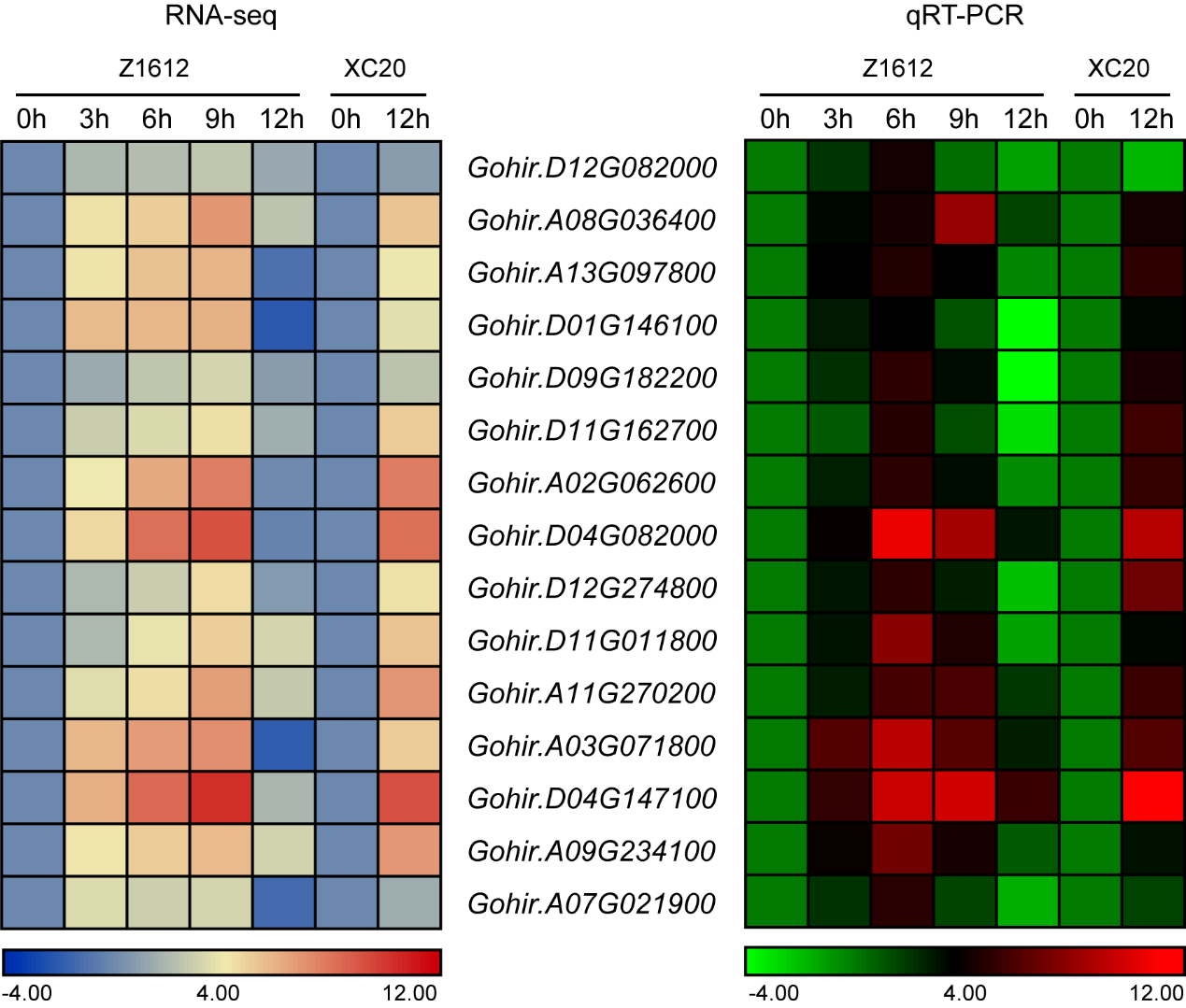
**

**S3 Fig.** **Correlation analysis of the RNA-seq data and qRT-PCR assay.** qRT-PCR detection of the selected 15 out of the 279 UDEGs was performed using the presented materials that were consistent with that for RNA-seq with three independent experiments. FPKM value was obtained from the RNA-seq data. Relative expression was determined by qRT-PCR, and was normalized using GhUBQ gene as internal control. The 0-h value was artificially set to 1, and the qRT-PCR-based heatmap was generated by R package. Gene numbers were shown in the middle of the diagram and the corresponding primers for qRT-PCR were listed in S3 Table .
