# Supplementary material for: Comparative transcriptome analysis of leaves during early stages of chilling stress in two different chilling-tolerant brown-fiber cotton cultivars
Source: PLoS One. 2021 Feb 9;16(2):e0246801. doi: 10.1371/journal.pone.0246801 (PMC7872267; doi:10.1371/journal.pone.0246801)
Supplement: S1 Table — (DOCX) [file pone.0246801.s004.docx]

**S1 Table. Length distribution of all unigenes.**

| **Samples** | **Clean reads** | **Clean bases** | **GC Content (%)** | **≥Q30 (%)** |
| --- | --- | --- | --- | --- |
| XC20-0-1 | 23,208,512 | 6,936,506,990 | 43.97% | 92.98 |
| XC20-0-2 | 22,020,026 | 6,581,983,450 | 44.58% | 94.31 |
| XC20-0-3 | 21,302,820 | 6,364,649,540 | 44.95% | 94.33 |
| XC20-12-1 | 23,474,461 | 7,026,553,182 | 45.47% | 93.45 |
| XC20-12-2 | 19,880,290 | 5,945,233,044 | 44.55% | 93.87 |
| XC20-12-3 | 19,704,002 | 5,891,170,832 | 45.47% | 94.05 |
| Z1612-0-1 | 30,205,826 | 9,042,461,612 | 44.85% | 94.21 |
| Z1612-0-2 | 26,382,297 | 7,887,066,402 | 44.79% | 94.03 |
| Z1612-0-3 | 23,849,501 | 7,135,334,486 | 44.81% | 93.81 |
| Z1612-3-1 | 31,755,644 | 9,494,019,588 | 44.18% | 94.44 |
| Z1612-3-2 | 25,646,623 | 7,663,023,174 | 46.61% | 94.21 |
| Z1612-3-3 | 33,777,618 | 10,102,856,732 | 44.68% | 92.21 |
| Z1612-6-1 | 22,598,951 | 6,760,919,808 | 44.77% | 94.50 |
| Z1612-6-2 | 23,097,150 | 6,910,449,428 | 45.11% | 94.15 |
| Z1612-6-3 | 25,583,017 | 7,649,947,604 | 44.92% | 94.13 |
| Z1612-9-1 | 21,685,824 | 6,485,502,920 | 44.11% | 92.69 |
| Z1612-9-2 | 21,973,070 | 6,572,156,580 | 43.93% | 93.15 |
| Z1612-9-3 | 20,373,102 | 6,090,202,206 | 45.57% | 93.77 |
| Z1612-12-1 | 23,679,270 | 7,087,858,224 | 44.95% | 94.01 |
| Z1612-12-2 | 19,970,463 | 5,972,310,228 | 45.21% | 94.04 |
| Z1612-12-3 | 23,223,958 | 6,942,594,610 | 44.31% | 94.01 |
