# Supplementary material for: Comparative transcriptome analysis of leaves during early stages of chilling stress in two different chilling-tolerant brown-fiber cotton cultivars
Source: PLoS One. 2021 Feb 9;16(2):e0246801. doi: 10.1371/journal.pone.0246801 (PMC7872267; doi:10.1371/journal.pone.0246801)
Supplement: S2 Table — (DOCX) [file pone.0246801.s005.docx]

**S2 Table. Details of functional annotation of all unigenes.**

| **Database** | **Number of genes** | **Percentage (%)** |
| --- | --- | --- |
| COG | 25754 | 35.45 |
| GO | 52754 | 72.61 |
| KEGG | 27002 | 37.17 |
| KOG | 39196 | 53.95 |
| NR | 56763 | 78.13 |
| Pfam | 52912 | 72.83 |
| Swiss-Prot | 67072 | 92.32 |
| NR | 71552 | 98.49 |
| Total | 72650 | 100 |
