# Supplementary material for: Comparative transcriptome analysis of leaves during early stages of chilling stress in two different chilling-tolerant brown-fiber cotton cultivars
Source: PLoS One. 2021 Feb 9;16(2):e0246801. doi: 10.1371/journal.pone.0246801 (PMC7872267; doi:10.1371/journal.pone.0246801)
Supplement: S3 Table — (DOCX) [file pone.0246801.s006.docx]

**S3 Table. Primers used in this work.**

| **Gene name** | **Primer (sequence orientation from 5’ to 3’)** |
| --- | --- |
| Gohir.D12G082000-F | AAAGCGAAGCTGAACGGAG |
| Gohir.D12G082000-R | AGGACCCCAGATGACGAAAC |
| Gohir.A08G036400-F | GGGGAGATATGGAGAAACGG |
| Gohir.A08G036400-R | TTGGTGGAGCTTGACCTGAC |
| Gohir.A13G097800-F | GGAAGGCGGGGATAAGAAA |
| Gohir.A13G097800-R | CTAATCTCACTGCCGTTTGG |
| Gohir.D01G146100-F | CCATCATCATCTGCTCCGTT |
| Gohir.D01G146100-R | TGAGGTTTTCTCCGTCTTCG |
| Gohir.D09G182200-F | AGGTGCTGAAACAGGTAAGGAG |
| Gohir.D09G182200-R | CAGGCGGCATTAACATTG |
| Gohir.D11G162700-F | CACTGACTGGCCGCAGTTTT |
| Gohir.D11G162700-R | CGTGTTGAGCAAGGTAGTAGGG |
| Gohir.A02G062600-F | TTGCTAATGGTGCTGGTGTC |
| Gohir.A02G062600-R | AGAAGAAGTCTTTGGGTGGC |
| Gohir.D04G082000-F | AAGTGCCCCTGAGCGAAAT |
| Gohir.D04G082000-R | GCTGAAGAGGGAACCGAAGT |
| Gohir.D12G274800-F | TCATCCGAGTTCCGACGAGT |
| Gohir.D12G274800-R | CATGGCAGTTGGAGGTAGGTAT |
| Gohir.D11G011800-F | GAACATTTACCTCCGCCTCG |
| Gohir.D11G011800-R | TCCCCAACTTCTGTCGTCTT |
| Gohir.A11G270200-F | GTGGCAGTGCTAAGAACAACAG |
| Gohir.A11G270200-R | CTCAACTTCCATCCACCCAT |
| Gohir.A03G071800-F | TGATGCCAAATGGGTTCG |
| Gohir.A03G071800-R | CACTGATTCCGCCAAGACT |
| Gohir.D04G147100-F | TTATCGCCCATCCACATCC |
| Gohir.D04G147100-R | TTCCGTAACCTTGCCGTCT |
| Gohir.A09G234100-F | AGCGACGGATTCGTGGAGTA |
| Gohir.A09G234100-R | GAAAACCCAGCCCAGTTCAT |
| Gohir.A07G021900-F | GGCATTGGAACAAGTCAACC |
| Gohir.A07G021900-R | GCAGCCATAACAGACACCCT |
| GhUBQ7-F | GAAGGCATTCCACCTGACCAAC |
| GhUBQ7-R | CTTGACCTTCTTCTTCTTGTGCTTG |
